# Stress Granules in the Post-transcriptional Regulation of Immune Cells

**DOI:** 10.3389/fcell.2020.611185

**Published:** 2021-01-14

**Authors:** Nicolas Curdy, Olivia Lanvin, Sarah Cadot, Camille Laurent, Jean-Jacques Fournié, Don-Marc Franchini

**Affiliations:** ^1^Cancer Research Center of Toulouse (CRCT), INSERM UMR 1037, CNRS ERL 5294, Toulouse, France; ^2^Université Toulouse III Paul Sabatier, Toulouse, France; ^3^Institut Universitaire du Cancer de Toulouse-Oncopole, Toulouse, France; ^4^Département de Pathologie, Centre Hospitalier Universitaire (CHU) de Toulouse, Toulouse, France

**Keywords:** stress granules (SG), immune cells, post-transcriptional regulation, translation, mRNA, RNA-binding proteins (RBP), integrated stress response (ISR), mTOR

## Abstract

Immune cell activation triggers transcriptional and translational programs eliciting cellular processes, such as differentiation or proliferation, essential for an efficient immune response. These dynamic processes require an intricate orchestration of regulatory mechanisms to control the precise spatiotemporal expression of proteins. Post-transcriptional regulation ensures the control of messenger RNA metabolism and appropriate translation. Among these post-transcriptional regulatory mechanisms, stress granules participate in the control of protein synthesis. Stress granules are ribonucleoprotein complexes that form upon stress, typically under control of the integrated stress response. Such structures assemble upon stimulation of immune cells where they control selective translational programs ensuring the establishment of accurate effector functions. In this review, we summarize the current knowledge about post-transcriptional regulation in immune cells and highlight the role of stress sensors and stress granules in such regulation.

## Introduction

Immune cell compartment is composed of two main cell types, the myeloid cells, such as macrophages or dendritic cells (DC), and the lymphoid cells, namely B and T lymphocytes. Macrophages and DC are key components of the innate immunity. They sense pathogenic threat and trigger adaptive immune response by presenting antigenic peptides to lymphoid cells. Upon antigen (Ag) recognition by their cell surface receptors [surface immunoglobulin (Ig) and T-cell receptor (TCR) in B and T cells, respectively], the lymphocytes become fully functional.

For all these immune cells, Ag stimulation triggers transcriptional and translational programs of differentiation, proliferation, and effector functions. Hence, the dynamics of immune response requires a tight control in gene expression, which can be achieved through post-transcriptional regulation. One important aspect of this regulation implies a decorrelation of transcriptional and translational activities for a given messenger RNA (mRNA) (de Sousa Abreu et al., 2009; Schwanhäusser et al., 2011; Buccitelli and Selbach, 2020). This regulation is mediated by multiple RNA-binding proteins (RBP) that perform their function on cis-acting elements typically lying on the 5′ and 3′ untranslated region (UTR) of mRNA (Gehring et al., 2017; Leppek et al., 2017; Mayr, 2017).

Actually, Ag stimulation and the ensuing cellular processes represent and are sensed as a physiological stress by immune cells. A general sensor of cellular stress is the integrated stress response (ISR), an evolutionary conserved signaling pathway that helps cells to adapt to environmental stress (Brostrom et al., 1996; Harding et al., 2000, 2003; Scheuner et al., 2001; Pakos-Zebrucka et al., 2016; Costa-Mattioli and Walter, 2020). At the center of ISR lies the eukaryotic initiation factor 2 (eIF2), which can be phosphorylated on its α subunit by four different kinases (Taniuchi et al., 2016), namely heme-regulated inhibitor (HRI or EIF2AK1) (Chen et al., 1991), protein kinase R (PKR or EIF2AK2) (Kostura and Mathews, 1989), PKR-like endoplasmic reticulum (PERK or EIF2AK3) (Harding et al., 1999), and general control non-repressible 2 (GCN2 or EIF2AK4) (Dever et al., 1992). Once phosphorylated, eIF2α prevents the formation of the ternary complex (eIF2:GTP:methionyl-initiator-tRNA) and consequently blocks translation initiation. Hence, activation of ISR globally inhibits translation while selectively preserving the expression of genes, including the transcription factor ATF4, to facilitate cell recovery (Wong et al., 1993; Brostrom et al., 1996; Harding et al., 2000; Lu et al., 2004; Schneider et al., 2020).

Importantly, this inhibition of translation caused by eIF2α phosphorylation seeds the formation of ribonucleoprotein complexes called stress granules (SG) (Kedersha et al., 2002; Panas et al., 2016; Protter and Parker, 2016). These structures sequester RNA upon stress and maintain a translation arrest until stress releases. SG can also form independently of eIF2α phosphorylation due to modifications in the eIF4F complex (comprising eIF4E, eIF4A, and eIF4G) (Emara et al., 2012; Fujimura et al., 2012; Panas et al., 2016). This complex is regulated by the mechanistic target of rapamycin (mTOR) kinase, acting as a metabolic sensor and regulator of translation (Ma and Blenis, 2009). When mTOR is inactivated, the eIF4E-binding protein 1 (4E-BP1) is no longer phosphorylated and prevents the formation of the eIF4F complex, which inhibits translation initiation and allows SG formation.

**Figure 1:**
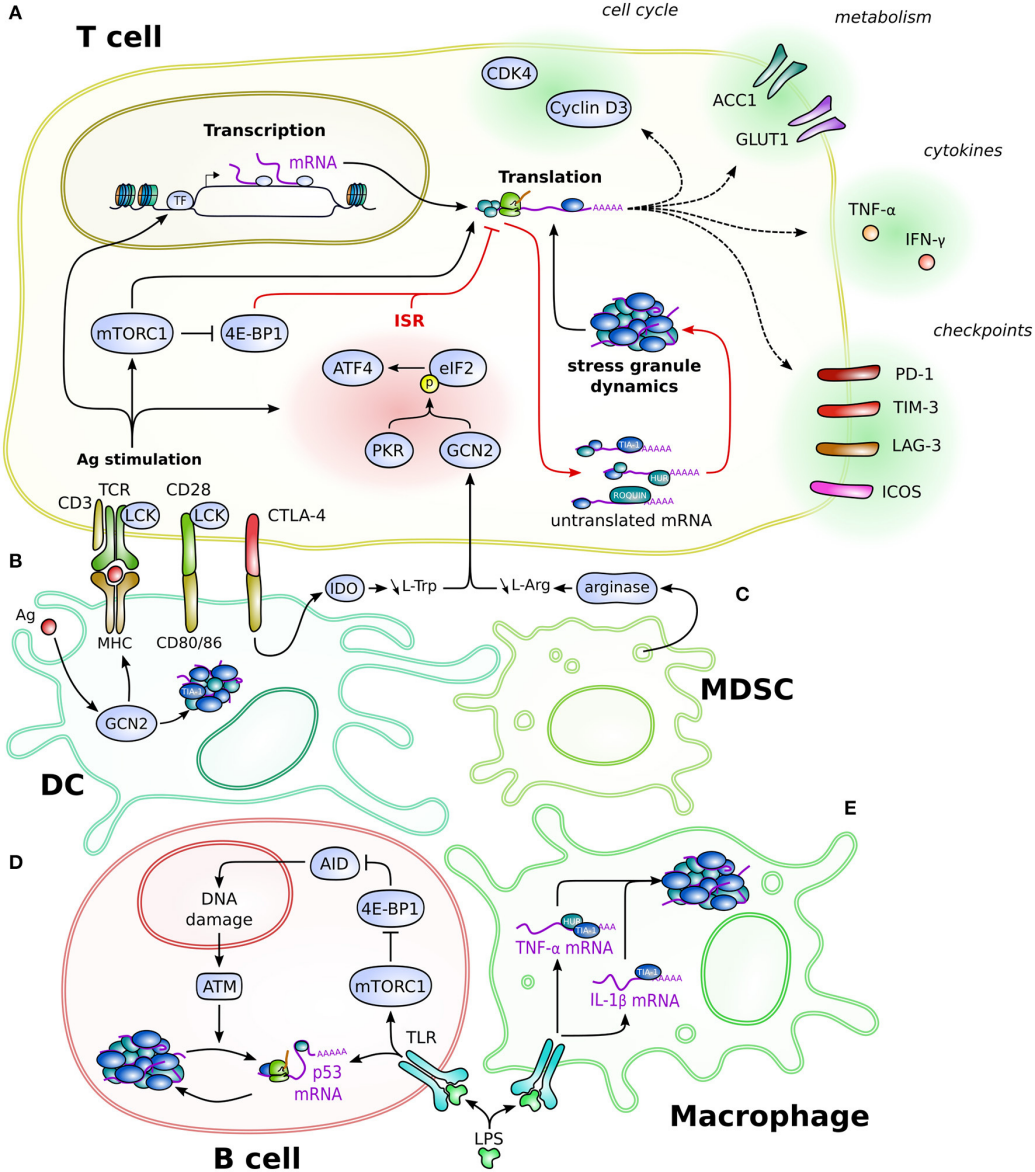
Schematic overview of the SG-based post-transcriptional regulation in immune cells. Depicted is the regulation in and connection between T cells **(A)**, DC **(B)**, MDSC **(C)**, B cells **(D)**, and macrophages **(E)**. Post-transcriptional mechanisms regulate different aspects of cellular biology, such as cell cycle, cytokine production, metabolism, or regulatory proteins like immune checkpoints, eventually impacting on cellular processes such as proliferation, differentiation, and effector functions. Red arrows specify the direct effector leading to translation inhibition and formation of stress granules. Dotted line arrows represent the product of translation that occurs once the mRNA is released from the post-transcriptional regulation. Cell-type-specific regulation is detailed in the text. Cells are not drawn at scale.

Understanding these post-transcriptional regulations and their mechanisms in immune cells is necessary and could help to improve treatments of autoimmunity and cancer. Here, we review the post-transcriptional regulation that are linked with SG in immune cells (Figure 1). Notably, this review will outline their involvement during T-cell activation, their control of cytokine expression, the influence of environmental cues, and how such mechanisms also control the expression of immune checkpoints (IC) in activated T lymphocytes. The following paragraphs will review the concurrent findings about the stress-dependent regulation of Ig diversification in B lymphocytes, of Ag presentation in DC, and finally of cytokine expression in myeloid cells.

## General Post-Transcriptional Regulation During T-Lymphocyte Activation

Prior to antigenic stimulation, T cells are actively maintained in a quiescent state due to post-transcriptional processes allowing high turnover and rapid degradation of RNA while maintaining a substantial amount of functional yet inactive ribosomal proteins (Ricciardi et al., 2018; Hwang et al., 2020; Wolf et al., 2020). Genome-wide “omics” analyses of transcriptome, translatome, and proteome during T-cell activation and differentiation pointed out a major role of post-transcriptional regulatory mechanisms (Hukelmann et al., 2016; Araki et al., 2017; Tan et al., 2017a,b; Howden et al., 2019) (Table 1). During these transitions, the regulation of ribosomal subunits expression and the activity of the mTOR complex 1 (mTORC1) signaling pathway are essential for shaping the proteomic landscape of T lymphocytes.

**Table 1:** Omics approaches suggesting post-transcriptional and translational regulation in immune cells.

**Omics analyses**	**Experimental models**	**Experimental conditions**	**Main regulatory elements**	**References**
Proteome Transcriptome	Mouse CD8^+^ cytotoxic T cells (P14 cells transgenic for TCR specific of LCMV gp33-41 glycoprotein)	Activated T cells	mTORC1 (control ~10% of total protein expression)	Hukelmann et al., 2016
Proteome Phosphoproteome	Mouse primary CD4^+^ T cells isolated from spleen or peripheral lymph nodes (PLN)	T-cell activation Naive → effector	mTORC1 (coordination of transcriptional, translational, and posttranslational programs)	Tan et al., 2017a
Proteome	Mouse CD4^+^ (OT-II; transgenic TCR reactive to ovalbumin) and CD8^+^ (P14) T cells	T-cell activation and differentiation Naive → effector	mTORC1 (cell context effects)	Howden et al., 2019
Proteome	Human primary CD4^+^ T cells isolated from PBMC	T-cell activation Naive → effector	Protein turnover and mTOR	Wolf et al., 2020
Transcriptome Translatome	Mouse CD8^+^ OT-I T cells (transgenic TCR specific of ovalbumin)	T-cell activation (Lck deficiency) Naive → effector	TCR signaling	Tan et al., 2017b
Transcriptome Translatome	Human primary CD4^+^ isolated from PBMC	T-cell activation Naive → effector	TCR activation and 4E-BP1	Ricciardi et al., 2018
Transcriptome Translatome	Mouse CD8^+^ (P14) T cells	T-cell activation Naive → effector → memory	TCR stimulation and mTOR	Araki et al., 2017
Transcriptome Translatome iCLIP	Mouse primary B cells isolated from spleen or PLN	Activated B cells—DNA damage	TIA-1 SG	Díaz-Muñoz et al., 2017

These features primarily depend on the TCR stimulation and its underlying signaling. This has been assessed by deleting *Lck*, one essential component of the TCR-mediated signal transduction complex, in CD8^+^ T cells (Tan et al., 2017b) (Figure 1A). In comparison with wild-type cells, *Lck*-deficient counterparts displayed a reduced mTOR signaling activity, reflected by the accumulation of unphosphorylated 4E-BP1, and failed to properly induce ribosome biogenesis. Consequently, overall protein synthesis was limited, thereby impairing the proliferative ability. The analysis of global RNA expression and their association with polysomes, which reflect the translation efficiency, in *Lck*-deficient T cells confirmed that one fourth of all mRNA were post-transcriptionally regulated (Tan et al., 2017b).

One important change that comes with immune cell activation and differentiation is metabolic reprogramming (Chapman et al., 2020). Metabolic analyses identified the clustering of T-cell subsets (Ricciardi et al., 2018) and defined the metabolic reprogramming triggered by T-cell activation (Ricciardi et al., 2018; Wolf et al., 2020) (Figure 1A). Interestingly, in naive CD4^+^ T cells, GLUT1 and ACC1 proteins, which, respectively, control glucose entry and fatty acid synthesis, are absent, while their corresponding mRNA are abundant and whose fraction is already associated with polysomes (Ricciardi et al., 2018). At this stage, 4E-BP1 is expressed and outnumbers eIF4E, and mTOR activity is low, indicating a control at the translation level (Ricciardi et al., 2018; Wolf et al., 2020). Interestingly, while TCR stimulation, which induces mTORC1 activation, promotes GLUT1 and ACC1 protein expression, there is still no correlation between the kinetics of appearance of mRNA vs. protein and neither with their association with polysomes (Ricciardi et al., 2018). This control of translation through the modulation of the eIF4F complex activity was further explored during T-cell polarization. Because mTORC1 has a dual translational regulatory activity, as repressor and inductor, this modulation could be necessary to selectively adjust translational programs to lineage identity (Hukelmann et al., 2016; Howden et al., 2019). In CD4^+^ T lymphocytes stimulated under T helper (Th) 17 polarization conditions, inhibition of eIF4E limits Th17 differentiation to promote Foxp3-expressing regulatory T-cell (Treg) development (Ricciardi et al., 2018). In agreement, genome-wide analysis of polysome-associated mRNA revealed that Treg have distinct translational abilities compared with non-Treg (Foxp3^−^) cells, mainly based on eIF4E expression, which was not detected in Treg (Bjur et al., 2013).

Thus, TCR stimulation controls selective translational activity through post-transcriptional mechanisms that maintain mRNA in complexes with repressive translational configuration and/or control their delivery to the polysomes.

## Control of Cytokine Expression by Stress-Dependent Mechanisms in T Lymphocytes

Analysis of translation regulation among the naive, effector (early and late, based on their proliferation status) and memory CD8^+^ T cells in mice during infection with the lymphocytic choriomeningitis virus (LCMV) clearly indicated a different translational ability during the activation and differentiation processes (Araki et al., 2017). Typically, *Ifng* mRNA levels increased after infection in early and late effector cells, while IFN-γ proteins peaked in early effectors and decrease in late effectors, correlating with the localization of the mRNA within the polysome fractions. In contrast, Granzyme B, a cytotoxic effector, is expressed in early effector cells but not in naive cells, which correlates with the level of mRNA expression and its association with polysomes. However, while the level of mRNA and the association to polysomes were similar in late effector and memory cells, these cells gradually lose the protein expression (Araki et al., 2017).

Thus, active post-transcriptional mechanisms control mRNA translation during T-cell activation by regulating the mRNA delivery to the polysomes and/or reconfiguring protein complexes controlling mRNA translation. Such modulation of translational regulatory complexes is controlled by stress sensor kinases, which are upregulated in stimulated T cells (Howden et al., 2019) (Figure 1A). In CD4^+^ T cells, the GCN2–eIF2α-ATF4 pathway favors Th1 over Th17 differentiation and is required for IFN-γ expression (Sundrud et al., 2009; Ravindran et al., 2016; Yang et al., 2018). In addition, it was suggested that *IFNG* mRNA regulates its own expression by activating PKR through a peculiar structure, called pseudoknot, in its 5′UTR (Ben-Asouli et al., 2002; Cohen-Chalamish et al., 2009). Binding of PKR on this pseudoknot results in eIF2α phosphorylation, which inhibits *IFNG* mRNA translation. Likewise, *TNFA* mRNA contains such pseudoknot in its 3′UTR (Osman et al., 1999). Recognition of this structure by PKR also leads to eIF2α phosphorylation. However, in this particular case, this signaling is required for efficient splicing of *TNFA* mRNA, suggesting an additional regulatory function for this pathway (Namer et al., 2017).

Under persistent antigen stimulation, mRNA encoding ribosomal proteins are still associated with active polysome fractions, indicating that stimulation maintains translation activity (Araki et al., 2017). In addition to ribosomal proteins, *Il4* mRNA is also relocated to active polysome fractions upon TCR re-engagement of primed CD4^+^ T lymphocytes under Th2 conditions (Scheu et al., 2006). Interestingly, T-cell priming triggers ISR, reflected by eIF2α phosphorylation, and SG, reflected by TIA-1-positive granule formation. Upon re-stimulation, the eIF2α phosphorylation decreases, which correlates with the increases of the translation activity. Moreover, deletion of TIA-1 bypasses the need of re-stimulation to produce the IL-4 protein, suggesting that the post-transcriptional regulation operating on *Il4* mRNA is mediated by the SG. However, TIA-1-positive granules persist upon re-stimulation, raising the question if *Il4* mRNA is directly regulated by SG, but anyhow indicating that SG is required throughout T-cell activation. Given the general variation in translation activity relative to T-cell activation, it is likely that the SG-dependent regulation is not restricted to cytokines (Scheu et al., 2006; Araki et al., 2017).

## Environmental Cues Influencing Stress-Dependent Regulation in T Lymphocytes

While antigenic stimulation induces important reorganization in T cells, other environmental signals can significantly influence T-cell biological functions (Figures 1A–C). Plasmacytoid DC from tumor-draining lymph nodes express indoleamine 2,3-dioxygenase (IDO), an enzyme involved in tryptophan starvation. IDO expression by plasmacytoid DC induces a GCN2-dependent proliferative arrest in T cells (Munn et al., 2005) (Figures 1A,B). Similar issues are induced by L-arginine starvation (Rodriguez et al., 2007), which can be caused by myeloid-derived suppressor cells that express high amounts of arginase (Figures 1A,C). T cell activated in such conditions failed to proliferate due to a lack of expression of cyclin D3 and cyclin-dependent kinase 4 (cdk4), which regulate cell cycle progression. Furthermore, the RBP HuR interacts with the 3′UTR of the *cyclin D3* mRNA to stabilize it (Rodriguez et al., 2010). Upon activation under L-arg deprivation, GCN2 is activated, which decreases the expression of HuR protein, but not the mRNA. Consequently, *cyclin D3* mRNA is no longer stabilized and does not proceed to translation. In addition to cyclin D3, a global translation inhibition due to L-arg deprivation was observed, suggesting that this regulatory mechanism is general (Rodriguez et al., 2010). Moreover, GCN2 activation is involved in tolerogenic mechanisms (Cobbold et al., 2009). While the proliferative arrest due to GCN2 activation promotes T-cell survival, this phenotype also depends on the mTOR pathway, as amino-acid (AA) starvation decreases 4E-BP1 phosphorylation. Along with GCN2, mTOR signaling provides a positive feedback for the generation of Treg. In addition, Treg promote T-cell proliferation arrest through a CTLA-4-dependent signaling that stimulates expression of AA-consuming enzymes, such as IDO, by DC (Cobbold et al., 2009) (Figures 1A,B).

The complexity of pathway interconnection could explain the role of GCN2 in controlling proliferation and trafficking of T lymphocytes upon stimulation (Van de Velde et al., 2016). Expression of ATF4 was partially dependent on GCN2 activity under AA deprivation and oxidizing environment in activated Th1-polarized CD4^+^ T cells (Yang et al., 2018). This ATF4 pathway could regulate mTORC1 activity under oxidative conditions and drive the metabolic reprogramming upon CD4^+^ T-cell stimulation (Yang et al., 2018), to allow proliferation and T-cell function (Ricciardi et al., 2018; Wolf et al., 2020).

## Control of Immune Checkpoint Expression by Stress-Dependent Mechanisms in T Lymphocytes

IC receptors play essential regulatory functions in immune cells and represent therapeutic targets for autoimmune diseases and cancers (Zhang and Vignali, 2016; Ribas and Wolchok, 2018). Among the different levels of regulation controlling IC expression, SG-based post-transcriptional mechanisms are implicated and represent important new therapeutic targets (Curdy et al., 2019; Gao et al., 2019) (Figure 1A).

In human CD4^+^ and CD8^+^ T lymphocytes, stimulation of resting cells induces the expression of SG markers and the formation of SG, which selectively control inhibitory IC expression (Franchini et al., 2019). This SG-dependent regulation relies on microtubules and the molecular motor kinesin1, with the 3′UTR of mRNA being essential, notably for PD-1 expression. When T lymphocytes are treated with microtubule-targeting drugs, the translation of PD-1, CTLA-4, TIM-3, LAG-3, TIGIT, and BTLA is inhibited (Franchini et al., 2019).

ROQUIN, a protein that plays an important role in preventing autoimmune diseases, is commonly found in SG. ROQUIN limits the inducible T-cell co-stimulator (ICOS) expression in CD4^+^ T cells and was found to bind directly to the 3′UTR of the *ICOS* mRNA, and both co-localize in SG (Yu D. et al., 2007; Athanasopoulos et al., 2010; Glasmacher et al., 2010). However, they are also found in processing bodies, another ribonucleoprotein complex involved in RNA degradation (Yu D. et al., 2007; Glasmacher et al., 2010), suggesting that in T lymphocytes, both types of granules are interconnected, as in other cell types (Protter and Parker, 2016). Moreover, the 3′UTR-dependent inhibitory regulation of ROQUIN was also observed with OX40, another stimulatory IC, suggesting a broader action of the SG-dependent regulation on stimulatory IC (Vogel et al., 2013).

## Stress-Dependent Regulation of Immunoglobulin Diversity in B Lymphocytes

Ig and TCR assembly relies on a series of genomic rearrangements that are intimately linked with DNA damage repair. In addition, mature B cells stimulated in germinal centers trigger the expression of the activation-induced deaminase (AID), which initiates class switch recombination and somatic hypermutation to increase Ig affinity and specificity toward the Ag (Franchini and Petersen-Mahrt, 2014). Following stimulation of primary murine B cells, TIA-1-positive SG are formed and sequester a substantial amount of mRNA, mainly through binding to their 3′UTR (Díaz-Muñoz et al., 2017) (Figure 1D). Among those mRNA lies *p53*, a key factor of DNA damage response. Upon etoposide treatment, an inhibitor of topoisomerase II that induces DNA double strand breaks used to mimic AID-induced DNA damage, ATM kinase signaling is triggered, releasing *p53* mRNA from SG and relocating it to active polysomes for translation, thereby promoting DNA damage repair (Díaz-Muñoz et al., 2017). Intriguingly, AID expression is controlled by 4E-BP1, and mTOR inhibition with rapamycin decreases AID protein but not its transcript (Chiu et al., 2019) (Figure 1D). Therefore, a sequential SG-dependent post-transcriptional regulation most likely underlies B-cell differentiation.

## Stress-Dependent Regulation of Antigen Presentation in Dendritic Cells

DC are able to sense pathogenic threat and to initiate adaptive immune response through Ag presentation (Pulendran, 2015). Following inoculation of the yellow fever virus vaccine, the kinase GCN2 is activated in DC (Ravindran et al., 2014) (Figure 1B). This activation strongly correlates with the subsequent T-cell response. Mechanistically, while GCN2 deficiency does not impair the secretion of inflammatory cytokines, it reduces autophagy-mediated antigen presentation and, consequently, impairs T-cell priming (Ravindran et al., 2014). In addition, stimulation of DC by vaccination induces the formation of TIA-1-positive SG (Ravindran et al., 2014). Although SG were shown to be crucial during innate antiviral immune response (McCormick and Khaperskyy, 2017), the real function of TIA-1 granules in that case was not established.

## Control of Cytokine Expression by Stress-Dependent Mechanisms in Myeloid Cells

Early studies revealed the importance of post-transcriptional regulation in the control of cytokine expression (Ivanov and Anderson, 2013). Upon stimulation, macrophages isolated from *Tia-1*-deficient mice have an increased proportion of *Tnfa* mRNA associated with polysomes and, consequently, overexpress TNF-α protein, which suggests a TIA-1-dependent control of mRNA translation (Piecyk et al., 2000). Interestingly, TIA-1-deficient lymphocytes do not overexpress TNF-α upon stimulation, indicating that this TIA-1-dependent regulation is cell-type-specific (Saito et al., 2001). Along with TIA-1, the RBP HuR and the protein steroid receptor coactivator (SRC-3) co-localize in SG following arsenite treatment, and they silence *Tnfa* mRNA translation in LPS-stimulated macrophages (Katsanou et al., 2005; Yu C. et al., 2007) (Figure 1E).

Pharmacological activation of the ISR pathway GCN2–eIF2α in LPS-stimulated macrophages controls IL-1β production by a mechanism involving TIA-1-positive SG (Battu et al., 2018; Naz et al., 2019) (Figure 1E). Likewise, deletion of *Gcn2* or *Atf4* in myeloid cells abrogates IL-1β expression (Ravindran et al., 2016; Yang et al., 2018). This influences CD4^+^ T-lymphocyte polarization in preventing Th17 differentiation and, therefore, synergizes with the intrinsic role of this ISR pathway in CD4^+^ T lymphocytes to favor Th1 differentiation (Sundrud et al., 2009; Ravindran et al., 2016; Yang et al., 2018). Modulation of this pathway in mice models allowed to reduce inflammation and protect against autoimmune disorders (Sundrud et al., 2009; Ravindran et al., 2016).

## Conclusion

External stimuli sensed by immune cells trigger specific functional programs that allow the completion of immune response and pathogenic clearance. It is now clear that the rapid variation in gene expression in stimulated immune cells is not only controlled at the transcriptional level but also implies post-transcriptional regulation, with SG emerging as a key regulator. The immune response relies on an intricate connection between the integration of environmental signals and the communication between cells, which leads to the specific development and differentiation of cell types. Although each cell type aims at providing a specific function, they all use similar regulatory mechanisms to control their protein expression. During immune cell activation, the ISR pathway, through the kinases that all converge on eIF2α, as well as the mTOR/4E-BP1 pathway, is activated. These pathways aim at SG formation for the control of mRNA translation.

The immune response, the underlying stress-dependent signaling pathways, and the biochemical properties of SG are all very dynamic. Such dynamic features would confer a greater versatility for immune cells to properly shape immune response. The intricate signaling network emphasizes the complexity of the control of immune response and suggests that each signal and resulting SG provide different regulatory outcomes. Whether each signaling pathway has regulatory functions in the different immune cell types, and to which extent, is still an open question. It will be therefore important to associate the dynamics of immune response with the activation of the different stress-dependent signaling pathways. Integrative single-cell molecular analyses are required for deciphering such interplay and to assign it within the immune cell population during the different cellular processes they undergo.

Proteome and transcriptome modulation ensuing from the integration of these distinct signaling pathways could influence SG constitution and function and dictate the dynamics of mRNA exchange within SG. It is now clear that the proteomic composition of SG is heterogeneous and depends on the nature of the stress and the cell type (Markmiller et al., 2018). Although TIA-1 was mainly used to identify SG in immune cells, one important aspect to resolve is whether different types of SG are formed in immune cells. Moreover, in addition to the primary stimulus, the influence of environmental factors, such as AA availability or cytokines, on SG formation and composition needs to be determined. This could provide targets to manipulate mRNA translation and will clarify the degree of heterogeneity in immune SG.

Besides SG proteomics, the evolution of the mRNA content depending on the cellular state of immune cells also needs to be addressed. While mRNA UTR appears important for SG targeting, they are themselves subjected to regulatory mechanisms (Mayr, 2016; Leppek et al., 2017). Cellular stresses modify 3′UTR mRNA features (Mizrahi et al., 2018; Zheng et al., 2018), which can eventually impact on RBP binding, such as TIA-1, and SG formation (Zheng et al., 2018). Similar 3′UTR modifications were observed in activated and proliferating lymphocytes (Sandberg et al., 2008; Gruber et al., 2014). While transcriptomic analyses are required to determine the mRNA content of SG and evaluate the regulatory function of SG, additional analyses that precisely characterize the mRNA features will be necessary.

The complex post-transcriptional regulation performed by SG warrants future studies to determine their role in the different functional programs of immune cells. Although proteomic and transcriptomic analyses of their SG will shed light on this regulation, another challenge will be to apply these studies to primary immune cells. To overcome these limitations, improvement and adaptation of single-cell analyses might turn out to be decisive for integrative elucidation of such regulation in immune cells. Their deciphering has the potential to lead to the development of novel therapeutic approaches for autoimmune disorders, viral infections, and cancer.

## Author Contributions

NC and D-MF conceptualized this review, decided on the content, and wrote the manuscript. NC and D-MF prepared the figures. OL, SC, CL, and J-JF revised this review. All authors approved the final version of the manuscript and agreed to be accountable for all aspects of the work.

## Conflict of Interest

The authors declare that the research was conducted in the absence of any commercial or financial relationships that could be construed as a potential conflict of interest.
